# A Cadaveric Case of Bilateral Adrenal Hyperplasia With Lung Cancer: A Paraneoplastic Syndrome?

**DOI:** 10.7759/cureus.59443

**Published:** 2024-05-01

**Authors:** Ganesh K Velayudham, Mohammed S Saqlain, Isabel Phillips, Juliette Saloway, Karuna Katti

**Affiliations:** 1 College of Medical and Dental Sciences, University of Birmingham, Birmingham, GBR

**Keywords:** case report, abdominal aortic aneurysm, lung neoplasm, paraneoplastic syndrome, adrenal masses, adrenal hyperplasia

## Abstract

Bilateral adrenal hyperplasia associated with primary lung cancer may arise from either an adrenocorticotropic hormone (ACTH)-dependent paraneoplastic syndrome or metastatic hyperplasia. Ectopic Cushing’s syndrome manifests as a paraneoplastic syndrome characterized by the secretion of ACTH from malignant cells. This secretion can in turn overstimulate the adrenal cortex, resulting in adrenal cortical hyperplasia followed by hypercortisolism. Though rare, lung cancer can metastasize to the adrenal glands bilaterally, occasionally resulting in hemorrhage causing a rapid increase in size. This cadaveric report aims to delineate an unusual case of metastatic lung cancer, presenting with bilateral adrenal hyperplasia and two abdominal aortic aneurysms (AAA). An 84-year-old white male cadaver was dissected during routine coursework. Dissection of the right lung revealed a pale-yellow mass measuring 10.0 x 7.4 x 7.0cm. An irregularly defined, necrotic, ulcerated lesion measuring 5.5 x 4.5 x 3.3cm was evident on the lateral surface of the left shoulder, consistent with a history of metastatic lung carcinoma. Upon abdominal dissection, bilaterally enlarged adrenal glands and two AAAs were observed. These AAAs measured 6.0cm and 11.0cm at their respective widest transverse diameters. The right and left adrenal glands measured 10.0 x 6.5 x 4.5cm and 7.3 x 4.7 x 3.5cm, respectively. We aim to discuss the possible pathophysiological correlation of these unusual findings in this cadaver.

## Introduction

Paraneoplastic syndromes are rare clinical manifestations associated with malignancy that are distinct from the effects of local invasion or metastases. These conditions often arise secondary to the ectopic secretion of hormones or functional peptides by tumor cells, which precipitates a cascade of metabolic changes. Paraneoplastic syndromes are often associated with lung cancers, and their manifestations are dependent on the histology of the primary tumor [1]. The unrestricted production of adrenocorticotropic hormone (ACTH) by malignant cells causes ectopic Cushing’s syndrome (ECS), which confers a poor prognosis [2]. Ectopic Cushing’s syndrome is largely associated with small-cell lung carcinoma (SCLC) and bronchial carcinoids, seen in 1% to 5% of SCLC [3]. Unabated ACTH secretion leads to hyperstimulation of the adrenal cortex, manifesting radiologically as adrenal cortical hyperplasia and subsequent hypercortisolism. Around 10% to 15% of ACTH-dependent adrenal cortical hyperplasia cases are due to ectopic secretion [4].

Patients with ECS may present with typical Cushingoid features, such as weight gain and purple striae. In severe cases, ECS can lead to a myriad of complications, such as hypokalaemia, hyperglycemia, diabetes, secondary hypertension, and thrombosis [5]. Hypertension is a major risk factor for the development, expansion, and rupture of abdominal aortic aneurysms (AAAs), alongside additional risk factors such as atherosclerosis, smoking, advanced age, and male sex [6,7]. Abdominal aortic aneurysms are a life-threatening condition defined as an abnormal dilation of the abdominal aorta with a diameter threshold of 3 cm [8]. To our knowledge, no case reports have documented lung cancer with secondary ECS, bilateral adrenal hyperplasia, and AAA. Reports documenting the photographic evidence of postmortem bilateral adrenal hyperplasia are also rare.

The aim of this work is, therefore, to present and discuss a cadaver with evidence of metastatic lung cancer and bilateral adrenal hyperplasia. Two large, unruptured AAAs demanded further scrutiny and speculation. Though histopathology was unavailable, such unusual findings during the dissection demanded further discussion on pathophysiological theories for their co-existence.

## Case presentation

The subject of this report is an 84-year-old white male cadaver with a past medical history of chronic obstructive pulmonary disease (COPD), left total hip replacement, right hernia repair, and an appendectomy in childhood. In 2021, he was diagnosed with metastatic lung cancer, showing liver and spleen metastases on CT imaging. He was subsequently referred for palliative care, and he passed away at his residence six to seven months after the diagnosis. The primary cause of death, as documented on his death certificate, was metastatic lung cancer. Chronic obstructive pulmonary disease and cerebrovascular disease were denoted as secondary causes. Due to confidentiality restraints, a detailed medical history was not available.

**Figure 1:**
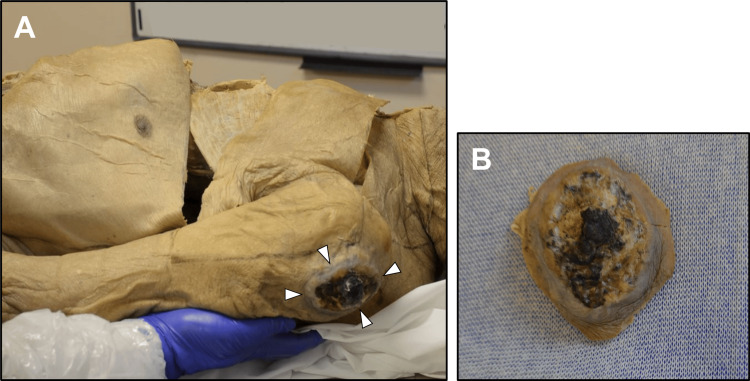
View of the left shoulder A: Lesion on the left shoulder. White arrowheads outline the irregularly defined, necrotic, and ulcerated lesion measuring 5.5 x 4.5 x 3.3 cm, present on the lateral surface of the left shoulder. B: Isolated lesion. Ulcerated and necrotic features are present.

On initial inspection, the cadaver exhibited severe cachexia. An irregularly defined, necrotic, ulcerated lesion measuring 5.5 x 4.5 x 3.3 cm was evident on the lateral surface of the left shoulder, confined to the skin and fascia with no invasion of the underlying deltoid muscle (Figure 1).

**Figure 2:**
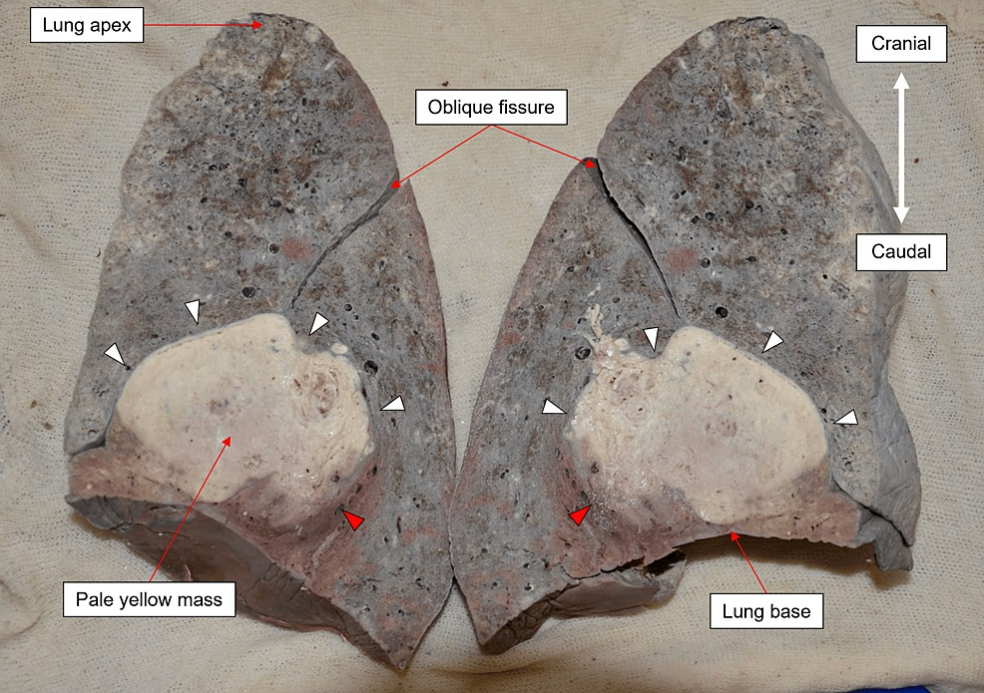
Right lung coronal section The image shows the tumor outlined by arrowheads, measuring 10.0 x 7.4 x 7 cm. White arrowheads demarcate the well-defined border and red arrowheads point to the infiltration.

Following the dissection of the thoracic cavity, the lungs were removed and weighed. The right lung weighed 1.28 kg, and the left lung weighed 1.03 kg. These were artefactually inflated weights due to the embalming process. Notably, the horizontal fissure of the right lung was absent; however, cadaveric studies have demonstrated that its presence is highly variable [9]. Consistent with the history, inspection of the right lung revealed a pale, yellow mass inferior to the hilum, indicative of a lung carcinoma section. Upon coronal sectioning of the right lung, the lesion measuring 10.0 x 7.4 x 7.0 cm was seen (Figure 2). It demonstrated a well-defined anterior border (white arrowheads in Figure 2) limited by the intact pleura of the oblique fissure, while the posterior border was poorly defined and irregular (red arrowheads in Figure 2), consistent with the macroscopic features of infiltration. No significant abnormalities were noted in the left lung.

Based on medical history, sections of the liver and spleen were examined to detect possible metastases; however, none were found upon gross examination. Additionally, due to the commonality of brain metastasis in lung cancer, the brain was also sectioned, but there was no evidence of metastatic spread [10]. Instead, hardening of the right vertebral artery was noted, aligning with a history of cerebrovascular disease.

**Figure 3:**
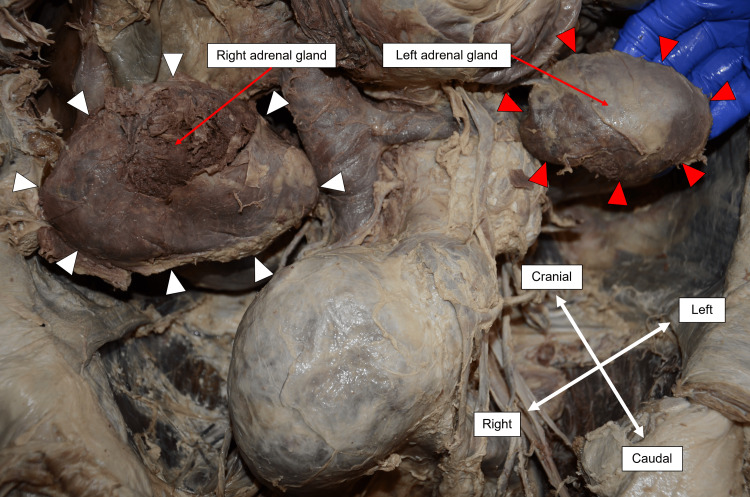
Right and left adrenal glands in situ White arrowheads outline the border of the right adrenal gland, measuring 10.0 x 6.5 x 4.5cm, and the red arrowheads outline the border of the left adrenal gland, measuring 7.3 x 4.7 x 3.5cm. The measurements refer to the length from left to right, the height from superior to inferior, and the depth from anterior to posterior, respectively.

**Figure 4:**
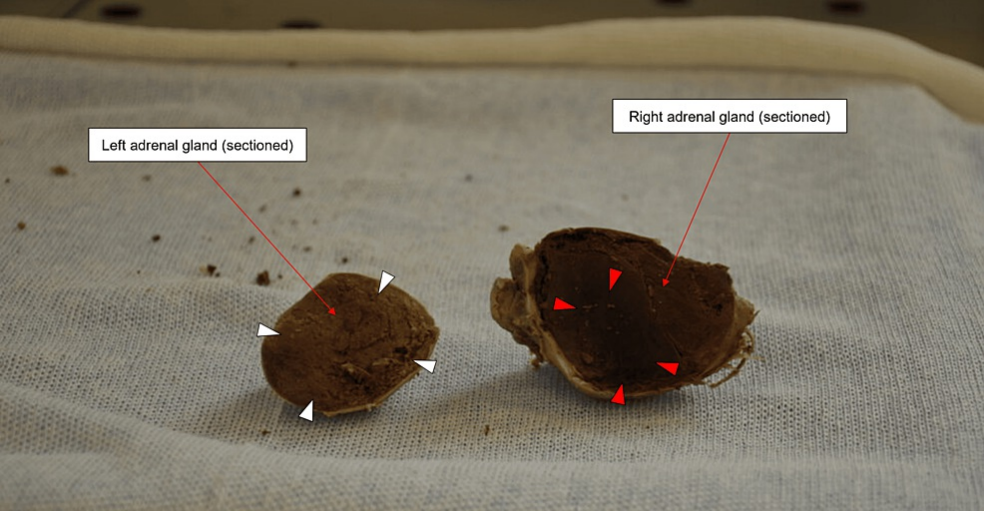
Sagittal sections of the right and left adrenal gland The adrenal glands were sectioned along their lengths from left to right. The white arrowheads indicate the normal appearance of adrenal parenchyma in the left adrenal gland, while the red arrowheads indicate evidence of interstitial hemorrhage in the right adrenal gland. No evidence of mass formation could be appreciated upon sectioning of both adrenal glands.

Before incising the abdomen for dissection, a midline laparotomy scar was identified, extending from the xiphisternum to the umbilicus. Subsequent abdominal dissection revealed bilaterally enlarged adrenal glands with a smooth, typical adreniform morphology. The dimensions of the right adrenal gland were 10.0 x 6.5 x 4.5 cm, and it weighed 130 g, while the left adrenal gland measured 7.3 x 4.7 x 3.5 cm, weighing 60 g (Figure 3). Upon sectioning, no evidence of mass formation could be appreciated, but the right adrenal gland showed signs of interstitial hemorrhage (Figure 4).

**Figure 5:**
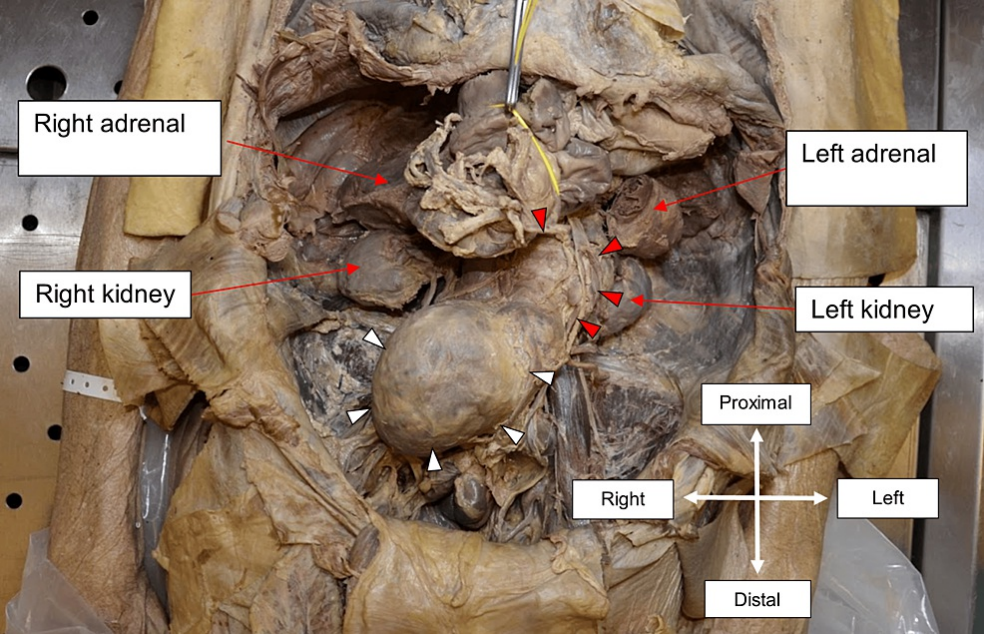
Two AAAs in situ The white arrowheads demarcate the larger aneurysm, measuring 11.0 cm. The red arrows show the smaller aneurysm, measuring 6.0 cm. Both were measured at their widest transverse diameter. AAAs: Abdominal aortic aneurysms

Furthermore, the examination revealed the presence of a double AAA (Figure 5). The proximal aneurysm extended into the left side of the abdominal cavity, positioned just superior to the level of the inferior mesenteric artery, while the distal aneurysm extended into the right side of the abdomen towards the right iliac fossa. The maximum transverse diameter of the proximal and distal aneurysms was 6.0 cm and 11.0 cm, respectively. This was measured using a string and ruler. The size of the distal aneurysm qualifies it as a giant AAA, which describes an AAA measuring between 10.0 cm and 13.0 cm in the maximum transverse diameter [11]. A stent was identified within the aorta from approximately the level of the renal arteries to the level of the aortic bifurcation, with minimal extension into each common iliac artery. A small branch measuring 3.0 mm in diameter was seen passing into the smaller aneurysm. The total length of the primary aortic stent was 14.9 cm.

## Discussion

In this 84-year-old male cadaver, several findings are notable: lung carcinoma, bilateral adrenal hyperplasia, two AAAs, and a lesion on the left shoulder. We propose the possibility of some pathophysiological connection between these occurrences. With lung cancer already confirmed in the cadaver’s medical history, the first novel discovery was the discovery of bilateral adrenal enlargement. The adrenal gland widths were 4.5 cm on the right and 3.5 cm on the left. Normal adrenal gland widths have been reported as 0.61 cm and 0.79 cm for the right and left adrenal glands, respectively [12]. Literature on adrenal hyperplasia does not typically describe glands of the magnitude displayed in this cadaver [13]. The unusually large adrenals suggest a significant underlying pathological process. While macroscopic examination alone cannot definitively establish the origin of the hyperplasia, we suggest it stemmed from adrenal cortical hyperplasia, possibly as part of a paraneoplastic syndrome linked to ectopic ACTH secretion by a lung tumor.

Adrenal cortical hyperplasia can develop during adulthood through either ACTH-independent or ACTH-dependent processes [4]. An ACTH-independent hyperplasia usually results from primary adrenal diseases, including functional adenoma and adrenal cortical carcinoma; however, these result in unilateral adrenal enlargement only [14]. The ACTH-independent bilateral hyperplasia is rare and is linked to germline mutations, which broadly result in adrenal hyperplasia with a micro- or macronodular appearance. However, considering the poor integrity of the glandular tissue upon sectioning, determining the degree of nodularity without histological examination was challenging. Nonetheless, given the rarity of ACTH-independent bilateral adrenal hyperplasia, an ACTH-dependent cause is more likely.

Cushing’s disease, caused by a pituitary corticotroph adenoma, is the most common cause of endogenous Cushing syndrome and concomitant adrenal cortical hyperplasia [4]. However, in this cadaver, the pituitary gland size was normal, thereby ruling out Cushing’s disease. Thus, ectopic ACTH secretion, often associated with lung and mediastinal masses, becomes the likely cause [15]. In conjunction with the established diagnosis of lung cancer, this points towards a paraneoplastic Cushing syndrome secondary to SCLC, as this is the predominant subtype responsible for ectopic ACTH secretion [3]. 

The progression of Cushing’s syndrome in such cases may differ from classical external manifestations due to rapid onset hypercortisolism leading to catabolic symptoms rather than typical Cushingoid features, such as central obesity, moon facies, and striae [5]. Despite weight gain being a common feature of Cushing’s syndrome, ECS is often associated with weight loss due to its catabolic effects and the advanced stage of malignancy at which it presents [15].

Excess glucocorticoids can lead to several complications, including hypokalaemia, hyperglycemia, and hypertension, partly through their mineralocorticoid effect [5]. Hypertension is a documented risk factor for AAA expansion [6]; it may be possible that hypertension, exacerbated by intense hypercortisolism, contributed to the growth of the two AAAs in this case. Correspondingly, reports have also suggested a possible link between aortic dissection and high cortisol levels secondary to Cushing’s syndrome [16,17]. The intact state and size of the AAAs suggest they were well-established before the onset of ECS. This is further supported by other risk factors for AAA within the history, namely old age, male sex, and smoking history (inferred from COPD history) [8].

A skin lesion on the left shoulder may represent a cutaneous metastasis from lung cancer. This is supported by its ulcerated appearance and parallels with documented cases; however, this cannot be confirmed without histology. Lung cancer metastasis to the skin is uncommon, occurring in approximately 1% to 12% of cases [18,19]. The shoulder is also a rare site for lung cancer metastases, with the most common sites reported to be the chest and abdomen [18]. Simsek et al. [20] describe a similar case of SCLC and a cutaneous metastasis to the left shoulder presenting as an indurated, erythematous, hard, nodular lesion. Although this case differs in that the lung cancer originated from the left lung instead, it holds parallels to ours.

Possible misdiagnosis of liver and splenic metastasis warrants consideration due to the proximity of hugely enlarged adrenal glands and the rarity of splenic metastasis [21]. We must also consider the possibility that the adrenal enlargement was subsequent to lung metastases and consequent hemorrhage. Adrenal metastasis is often secondary to lung cancer. Although adrenal metastasis is not uncommon, it is generally only noticed incidentally or postmortem, as it rarely presents clinically [22]. This may explain the absence of this finding in the medical history. On gross examination, the adrenals showed diffuse hyperplasia and signs of interstitial hemorrhage were seen in the right adrenal (Figure 4). Non-traumatic adrenal hemorrhage is a rare occurrence and can be caused by many factors, like disseminated intravascular coagulation, bacterial infection, and malignancy. While initial metastasis would not have resulted in such gross enlargement of the adrenals, subsequent interstitial hemorrhage may have resulted in their expansion, as seen in a case by Tatineni et al. [23].

We propose a picture of either a lung cancer-related paraneoplastic syndrome causing bilateral adrenal hyperplasia or metastatic hemorrhagic bilateral adrenal hyperplasia secondary to lung cancer. Both are rare occurrences. The limitations of this study include inaccessible medical records, a lack of laboratory investigation, and histological confirmation.

## Conclusions

We present a cadaveric case of lung cancer with associated findings of bilateral adrenal hyperplasia. Bilateral adrenal hyperplasia may be due to ECS secondary to lung cancer or metastasis from the lung primary. However, histopathology and biochemistry analyses are required for confirmation. While we agree with the cause of death as stipulated on the death certificate, we can, however, add a potential finding of paraneoplastic syndrome in the form of bilateral adrenal hyperplasia. We were unable to demonstrate liver or spleen metastases as per clinical history, but the primary tumor’s macroscopic features, along with the necrotic skin lesion, would support its malignant, infiltrative, and metastatic features.
